# Influence of Positive and Threatening Awe on Pro-Environmental Behavior: The Mediating Role of Connection to Nature

**DOI:** 10.3390/bs15050686

**Published:** 2025-05-16

**Authors:** Jia Liu, Yongquan Huo, Jing Wang, Yangyu Du, Xiangyu Li

**Affiliations:** 1 College Students’ Psychological Assistance and Research Center, Xi’an Conservatory of Music, Xi’an 710061, China; liujia@xacom.edu.cn; 2 School of Psychology, Shaanxi Normal University, Xi’an 710062, China; hyq@snnu.edu.cn (Y.H.); jingwang@snnu.edu.cn (J.W.);; 3 School of Aircraft, Xi’an Aeronautical Institute, Xi’an 710077, China; 4 School of Management, Xi’an Jiaotong University, No. 28, Xianning West Road, Beilin District, Xi’an 710049, China

**Keywords:** awe, pro-environmental behavior, ecotourism, connection to nature

## Abstract

This study aimed to verify the effect of positive awe and explore the effect of threatening awe on promoting pro-environmental behavior, as well as examine the role of connection to nature in this relationship. An online experiment (Experiment 1) and a laboratory experiment (Experiment 2) were conducted in two different samples to replicate the results. The results of Experiment 1 demonstrated that both positive and threatening awe promoted participants’ willingness to pay a premium for ecotourism, and the connection to nature played mediating roles in these processes. The results of Experiment 2 demonstrated that positive awe improved participants’ intention to conduct a series of pro-environmental behaviors, and the connection to nature played a mediating role, while threatening awe did not have this effect. These findings suggest that positive awe can encourage people to conduct more pro-environmental behaviors, while threatening awe can encourage pro-environmental behaviors in the form of financial support, and the feeling of connection to nature is one of the psychological mechanisms in these processes. The results of the present research not only enrich the literature related to both awe and pro-environmental behavior, but also provide managerial implications for policy makers and ecotourism operators.

## 1. Introduction

Global warming and severe weather have already seriously affected the lives of contemporary populations, and worsening environmental problems will have negative effects on future generations (Zaman & Moemen, 2017). Thus, there is a growing interest in studying environmental behaviors and the factors that influence them (e.g., Ertz et al., 2016; Wang & Huo, 2022).

Pro-environmental behavior (PB) refers to behaviors that are intended to prevent harm to the environment, or even to benefit it (Steg & Vlek, 2009; Stern, 2000). Consumers’ pro-environmental behaviors in daily household activities (such as recycling) and product or service choice (such as travel to nature) can have direct environmental impacts (Chiu et al., 2014; Stern, 2000). Therefore, it is necessary to focus on the factors that can promote private-sphere PB and environment-friendly traveling to provide possible intervention ways promoting the tourists’ pro-environmental behaviors.

The emotional factors influencing PB such as awe has not received sufficient attention in the tourism literature. Only in recent years have some related studies appeared in the fields of psychology and tourism (e.g., Pearce et al., 2016; Xu & Hu, 2024). However, most existing research focused on the influences of tourists’ awe-related experiences on post-travel PB (e.g., Xu & Hu, 2024), while less of them explored the effects of inducing awe on the PB of the potential tourists. In addition, few of them examined the same effect of the other type of awe—“threatening” awe (Pérez et al., 2023).

The present research aimed to verify the effects of the induced positive awe on promoting environment-friendly traveling and other private-sphere PBs. Additionally, the present research explored whether the effects of induced threatening awe on PB are consistent with those of positive awe, as well as the role of connection to nature in these relationships. This study not only enriches the theories related to both awe and pro-environmental behavior, but also provides managerial guidelines for policy makers and ecotourism operators.

## 2. Literature Review and Research Hypothesis

### 2.1. Awe and PB

Behaviors with the intention to protect or benefit the environment are regarded as PB (Steg & Vlek, 2009; Stern, 2000). According to the impact sphere of the behaviors, they can be classified into private and public sphere behaviors (Stern, 2000). Private-sphere behavior encompasses the everyday actions individuals take within their personal lives, including the acquisition, consumption, and disposal of personal and household items that contribute to environmental impact (Stern, 2000), while public-sphere behavior pertains to actions taken in the public domain, such as advocating for environmental policies or engaging in environmental protests (Stern, 2000). Researchers studying psychology and consumer behaviors have focused mainly on PB in the private sphere, such as buying environment friendly products, recycling, using public transportation, etc., (e.g., Ramkissoon, 2020; Wang & Huo, 2022), among which, the choice and purchase of the recreational travel mode can have significant influence on the environment (Chiu et al., 2014; Stern, 2000).

Ecotourism denotes a form of travel that takes individuals to relatively pristine or minimally impacted areas, where they engage in the appreciation and study of local landscapes and wildlife, all while minimizing their influence on the environment and the lives of local communities (Chiu et al., 2014). Given the need to protect the ecological environment, ecotourism tends to demand more from consumers, such as having to spend more money (Lu et al., 2016). Ecotourism is beneficial to both the economy and environmental protection (Chiu et al., 2014), which can be an important development direction of the tourism industry and has now gained the attention of many researchers (e.g., Lu et al., 2016). Therefore, investigating factors that promote individuals’ willingness to pay more for ecotourism can offer valuable empirical insights into fostering environmental sustainability. Accordingly, Experiment 1 used the propensity to pay a premium for ecotourism as an indicator of PB, which is a typical private-sphere PB, and Experiment 2 examined on a more comprehensive index of private-sphere PB in daily lives.

Previous studies have identified several factors influencing PB, such as contextual factors, attitudes toward PB, values of an altruistic or egoistic nature (Ertz et al., 2016; Stern, 2000). Some researchers have also begun to focus on the influence of irrational factors, such as emotions, on PB.

Awe is a mixed emotion evoked by things bigger than oneself, and needs to modify existing schemas to fit new circumstances (Chirico & Yaden, 2018; Keltner & Haidt, 2003). It can be felt when facing magnificent natural scenery or confronting an admirable figure (Keltner & Haidt, 2003). From the perspective of functionalism, awe may initially arise from one’s feelings about their shelter (Chirico & Yaden, 2018). Prospect and refuge theory (Appleton, 1996) believes that in ancient times, an ideal shelter fulfilled two basic human needs: safety and vantage. That is to say that one side of the shelter can protect people from danger, and the other side has a wide view. Usually, this kind of terrain is represented by tall canyons, which is a typical scene that can induce awe (Chirico & Yaden, 2018). Therefore, awe is a signal that an individual is in a safe place, which can make people transcend themselves, thus turning their attention to others and conducting pro-social or pro-environmental behaviors (Chirico & Yaden, 2018). If people conduct pro-environmental behavior at that time, it will make the environment of this world better, thus further providing protection and support for human beings and being conducive to people’s survival, thus forming a virtuous circle.

The feeling of awe includes not only wonder and amazement, but also fear and nervousness (Chaudhury et al., 2021; Liu et al., 2023). Thus, awe can be recognized as positive awe or threatening awe according to the valence (Liu et al., 2023; Nakayama et al., 2020). Positive awe is often considered to have the characteristics of typical positive emotions, while threatening awe can make people feel like they have less control over the situations, and in turn have a feeling of powerlessness (Liu et al., 2023; Nakayama et al., 2020). Inducing positive awe has been found to improve individuals’ well-being, but threatening awe did not have this effect (Liu et al., 2023; Nakayama et al., 2020). Although the different characteristics of positive and threatening awe indicated that they might have different effects on PB, the empirical results have shown that both valences of awe might promote PB similarly, despite the fact that they may do this through different mechanisms (Sun et al., 2021; Yang et al., 2018). However, research on threatening awe and PB is far from sufficient, and the effect of the connection to nature in this relationship also requires exploration (Nakayama et al., 2020; Pérez et al., 2023).

Our study aims to examine the influence of awe—both positive and threatening—on pro-environmental behaviors, specifically the willingness to pay a premium for ecotourism (Experiment 1) and the intention to conduct private-sphere PB (Experiment 2), through two experimental investigations. According to the theoretical analysis and existing research, both positive awe and threatening awe should be able to promote pro-environmental intentions or behaviors. Thus, we proposed two hypotheses for both experiments:

**H1.**  *Inducing positive awe (vs. control group) promotes PB (willingness to pay more for ecotourism for Experiment 1; intention to conduct pro-environmental behaviors for Experiment 2).*

**H2.**  *Inducing threatening awe (vs. control group) promotes PB (willingness to pay more for ecotourism for Experiment 1; intention to conduct pro-environmental behaviors for Experiment 2).*

### 2.2. The Mediating Role of Connection to Nature

Connection to nature refers to the degree to which individuals feel emotionally bonded to and have a sense of belonging within the natural environment (Mayer & Frantz, 2004; Tam, 2013). Among various instruments developed to assess one’s level of connection to nature, one of the widely used measurements is the Connection with Nature Scale (CNS; Mayer & Frantz, 2004). Besides considering connection to nature as a trait, researchers (Mayer et al., 2009) also proposed the state version of CNS as a state to assess the degree of connection to nature at a given point. In this study, state CNS will be used as the level of state connection to nature after emotion inducting manipulation.

Connection to nature has been considered and proved to be an important predictor of PB. The perspective of constructionist self-theory states that the concept of self is constructed by the interaction between the subject and the object (including nature); it extends the concept of self to ecology and regards nature as a part of self (Naess, 1985; Tam, 2013). People who regard nature as a part of themselves and feel connected with nature may think it necessary to protect nature from harm, and engage in PB (Bragg, 1996; Tam, 2013). Conversely, if people have not included nature in their own identity and closely connected themselves with nature, one might destroy the natural environment (Bragg, 1996; Tam, 2013). Existing studies have also confirmed the significant role of connection to nature in promoting PB. A meta-analysis examining 37 studies showed that participants with a stronger connection to nature reported higher levels of self-engagement in PB (Whitburn et al., 2020).

Awe is a type of self-transcendent experience which might increase the feeling of connection. According to Yaden et al.’s (2017) theoretical framework, the intensity spectrum of self-transcendent experience is a unitary continuum with different intensities of weakened self-awareness (annihilational component) and a stronger sense of connection (relational component) (Chirico & Yaden, 2018; Yaden et al., 2017). In addition to the emotion of awe, it also includes gratitude, appreciation, etc. As a self-transcendent emotion, awe often makes individuals pay less attention to themselves and turn to other broader things around them, increasing the sense of connection with one’s surroundings, including elements of nature (Yaden et al., 2017). Empirical research also supports the concept that “connectedness” is a key element in awe experiences (Yaden et al., 2019). Researchers have found that experiencing awe is positively correlated with higher levels of connection to others or nature (Van Cappellen & Saroglou, 2012; Yang et al., 2018). It should be mentioned that previous studies have also found that inducing positive awe can improve PB, and connection to nature played a mediating role in this process (Yang et al., 2018).

The other type of awe—threatening awe—has a flavor of fear (Chaudhury et al., 2021; Pérez et al., 2023), which might lead to avoidance motivation (Gable & Harmon-Jones, 2010), and further reduce the sense of connection. Thus, the effect of threatening awe on connection to nature and PB is likely to be complicated. However, threatening awe can be induced by natural disasters or extreme weather scenes. This might make people become more aware of how humans and nature interact with each other and strengthen their bond with the natural world (Zhang et al., 2014). Combined with the aforementioned idea that awe is a self-transcendent emotion which could improve a sense of connection to nature (Yaden et al., 2017), we hypothesized in both experiments that:

**H3.**  *Inducing positive awe (vs. control group) promotes participants’ feelings of connection to nature.*

**H4.**  *Inducing threatening awe (vs. control group) promotes participants’ feelings of connection to nature.*

In summary, since awe can enhance the sense of connection to nature, and the sense of connection to nature can effectively predict individuals’ PB, we hypothesized that connection to nature plays a mediating role in the relationship between inducing awe and PB. Thus:

**H5.**  *Participants’ feeling of connection to nature mediates between positive awe-inducing (vs. control group) and PB (willingness to pay more for ecotourism for Experiment 1; intention to conduct pro-environmental behaviors for Experiment 2).*

**H6.**  *Participants’ feeling of connection to nature mediates between threatening awe-inducing (vs. control group) and PB (willingness to pay more for ecotourism for Experiment 1; intention to conduct pro-environmental behaviors for Experiment 2).*

### 2.3. Present Research

Although researchers have examined the emotional factors influencing PB, fewer of them explored the effects of inducing awe on PB of the potential tourists. In addition, few of them focused on the other type of awe—threatening awe (Pérez et al., 2023).

The present research aimed to verify the effects of the induced positive awe on PB, and explored whether the effects of induced threatening awe on PB are consistent with those of positive awe, as well as the role of connection to nature in this relationship. Two experimental studies were conducted using different awe-inducing manipulations and different indicators of PB in two different samples to replicate the results logically. Accordingly, Experiment 1 sought to investigate the impact of awe on the propensity to pay a premium for ecotourism as an indicator of PB (a typical private-sphere PB), and Experiment 2 examined on a more comprehensive index of private-sphere PB to further replicate the result of Experiment 1.

## 3. Experiment 1: Awe and the Willingness to Pay More for Ecotourism

### 3.1. Method

#### 3.1.1. Participants

Two hundred adults living in mainland China were recruited through a website named Questionnaire Star (https://www.wjx.cn/), which pays people small amounts of money to do tasks. After removing invalid questionnaires, such as those that failed to recall an awe-based memory or failed the attention test, the final participants were 166 adults (82 male) aged 19–64 years (*M*_age_ = 30.86, *SD* = 7.43). The socio-demographic profiles of the participants can be seen in Table S1 of the Supplementary Materials. All participants completed the experiment on the internet in exchange for a payment.

#### 3.1.2. Materials

Dispositional Connection to Nature. The initial level of dispositional connection to the nature of the participants was assessed using the Inclusion of Nature in the Self scale (INS; Schultz, 2002). It includes only one item which asked participants to select from 7 pairs of circles that overlap to symbolize the relationship between “Self” and “Nature”. A higher score (from 1 to 7) indicates a higher level of dispositional connection to nature. The INS scale has been widely used (Schultz et al., 2004).

Awe-Inducing Manipulation. We used the recalling and writing tasks to induce certain emotions (positive awe, threatening awe, and neutral emotion) (Gordon et al., 2017; Pérez et al., 2023). According to prior research (Gordon et al., 2017; Pérez et al., 2023), natural scenery or natural disasters could induce the feeling of awe in different valences; therefore, participants in the awe conditions (both positive and threatening) were instructed to recall a nature-related memory. Participants in the control condition were instructed to recall the last time they went shopping at a supermarket. All participants were informed to recall these experiences and write at least five sentences about how they felt and what they did. To ensure the quality of the recall and writing task, the task was presented on a stand-alone webpage, and the minimum answer time was set to three minutes before they could continue to complete the content that followed. The detailed instructions can be seen in the Supplementary Materials.

Manipulation Check. After awe-induced manipulation, participants reported their feelings of several emotions at that moment. The average of awe, wonder, and amazement represented the awe score (α = 0.81); the average of fear, anxiety, and nervousness represented the fear score (α = 0.85); the average of amusement, joy, and warmth represented the positive affect score (α = 0.84); and the average of anger, sadness, and shame represented the negative affect score (α = 0.52) (Gordon et al., 2017). Each item was assessed using a 7-point Likert scale, ranging from “1-not at all” to “7-very much”.

The measurements of the Manipulation Check were used in both experiments. CFAs were conducted to verify the validity of the measurements. As seen in Table 1, except for the negative affect in Experiment 1, the Cronbach’s alphas and the CR values of all measurements were all above 0.8 in both experiments, the AVE values were all above 0.5, indicating that the reliability and validity of the measurements were acceptable (Bagozzi & Yi, 1988). The items and their factor loadings were all acceptable (Table S2, Supplementary Materials). Although the factor loading of the item of “shame” in the index of negative affect in Experiment 1 was relatively low (0.188), it was significant at the level of 0.05 (Table S2). Thus, we included this item in the manipulation check in both experiments. In addition, we also conducted the manipulation check without this item and found that the results remained the same, the Cronbach’s α of negative affect in Experiment 1 was 0.53, the CR was 0.63, and the AVE was 0.47. As the items of negative affect were used as manipulation check for emotional induction which was not used in the subsequent data analysis, we think it was acceptable (Bagozzi & Yi, 1988).

The State of Connectedness to Nature. The state CNS developed by Mayer et al. (2009) was used. This scale was modified from the trait version to assess an individual’s sense of connection to nature at that moment. It comprises 13 items, such as “At the moment, I am feeling that the natural world is a community to which I belong” (Mayer et al., 2009). Each item was assessed using on a 7-point Likert scale, ranging from “1—strongly disagree” to “7—strongly agree”. The average score was calculated to represent feeling of connection to nature at that time. This version of the scale has been widely used among Chinese populations (Wang & Huo, 2022).

The state CNS was used in both experiments. As seen in Table 1, the reliability and validity of the scale were all acceptable (Bagozzi & Yi, 1988). The items and their factor loadings were all acceptable (Table S2). Although the 12th item’s factor loadings were relatively low in both experiments (0.306 and 0.191, respectively), they were significant at the level of 0.05 (*p*s < 0.05; Table S2). Thus, we included this item in the following analysis. In addition, we also conducted all the analyses in both experiments without this item and found that the pattern of results remained the same.

Willingness to Pay More for Ecotourism. Adopting existing research paradigms (Lu et al., 2016), the participants were presented with an introductory text about ecotourism so that they could understand the significance and role of ecotourism in protecting the ecological environment (Chiu et al., 2014). Subsequently, the participants were informed to evaluate their willingness to pay more for ecotourism in future travel based on the environmental protection effect of ecotourism. It comprised five items (Table S2), such as: “How willing would you be to go on a more expensive holiday to reduce pollution?”. Each item was assessed using a Likert scale, ranging from “1—strongly unwilling” to “7—strongly willing”. The average was taken as the participant’s pro-environmental behavior. The psychometric properties of the scale were acceptable (Table 1 and Table S2).

Demographic Variables. Finally, the demographic variables of the participants were collected, including sex, age, living location, educational background, occupation, and monthly income.

#### 3.1.3. Procedure

Participants completed the experiment through an online questionnaire survey platform called Questionnaire Star (https://www.wjx.cn/). They completed the INS scale after providing informed consent. Thereafter, they were randomly assigned to one of three conditions and informed to recall their experiences. Participants were then informed to complete a manipulation check of the inducing task and the state CNS. After that, they were informed to evaluate their willingness to pay for ecotourism. Lastly, participants completed a demographic questionnaire.^1^

#### 3.1.4. Statistical Analysis

For both experiments, we used AMOS 24.0 to conduct CFAs, SPSS 25.0 to conduct descriptive statistics and ANOVAs, and the PROCESS 2.16 macro to conduct mediation analysis (Hayes, 2013).

### 3.2. Results

Manipulation Check. Firstly, two psychological researchers (the first author and the third author) evaluated the content of the recalling and writing task independently, and resolved disagreements through discussion. We excluded participants with invalid writing content such as writings that did not make sense, writings expressing a failure to recall a memory of awe, or writings not mentioning either of the awe-related affect (awe, wonder, and amazement).

Secondly, a one-way ANOVA was conducted to compare the emotions induced across the conditions. The results revealed that both awe groups reported significantly higher levels of awe compared to the control group. Furthermore, the threatening awe group reported significantly greater fear and negative affect than those in the other conditions; and the positive awe group reported significantly higher levels of positive affect than the other groups. Thus, the recalling and writing tasks effectively induced different valences of awe (Table 2).

Preliminary Analysis. ANCOVAs were conducted to compare the state CNS and willingness to pay for ecotourism after inducing different emotions across conditions. Considering the diversity of the sample and the possible influence of the original trait connection on the nature of the participants, we used participants’ INS, sex, age, and monthly income as covariates in this analysis and further analysis. As seen in Table 2, both awe groups scored greater state CNS than those in the control condition. The results supported H3 and H4. Regarding willingness to pay for ecotourism, the scores of the two awe conditions were significantly higher than those of the control condition. The results supported H1 and H2.

Mediation Analysis. Model 4 of the PROCESS macro (Hayes, 2013) was used to test the mediation effect of state connection to nature in the relationship between emotional priming and the willingness to pay for ecotourism. Following the procedure of mediation analysis (Hayes & Preacher, 2014), we entered the awe condition as categorical independent variable, state connection to nature as mediator, and willingness to pay for ecotourism as dependent variable. We also entered sex, age, monthly income, and dispositional nature connectedness (INS) as control variables.

The results of the omnibus test showed that the total effect was significant (*F*(2, 159) = 11.60, *p* < 0.001); however, the direct effect was not (*F*(2, 158) = 2.19, *p* > 0.05). The omnibus mediation effect was significant (95% Bootstrap confidence interval [0.04, 0.17], excluding 0). Thus, we conducted the relative mediation analysis.

**Figure 1:**
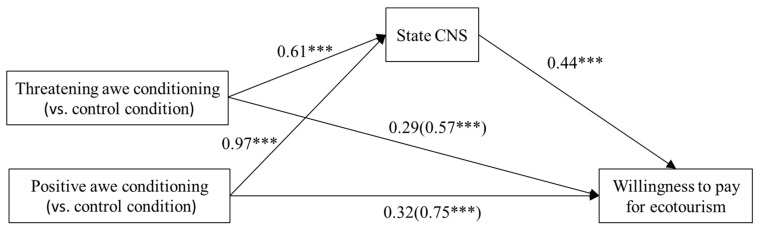
Mediation model of Experiment 1 (Nonstandard results). Note. Controlling for sex, age, monthly income, and INS. *** *p* < 0.001.

Relative to the control condition, the relative indirect effect of threatening awe conditioning was significant, with a 95% Bootstrap confidence interval of [0.11, 0.50], excluding 0 (Figure 1). This effect had an effect size of 0.27 (*SE* = 0.10). Additionally, the relative total effect was significant, with an effect size of 0.57 (*p* < 0.001). After controlling for the relative indirect effect, the relative direct effect was found to be non-significant (*p* > 0.05). The relative indirect effect explained 47.37% (0.27/0.57) of the relative total effect (Figure 1). The results supported H6.

Relative to the control condition, the relative indirect effect of positive awe conditioning was significant, with a 95% Bootstrap confidence interval of [0.23, 0.69], excluding 0 (Figure 1). This effect had an effect size of 0.43 (*SE* = 0.12). Additionally, the relative total effect was significant, with an effect size of 0.75 (*p* < 0.001). After controlling for the relative indirect effect, the relative direct effect was found to be non-significant (*p* > 0.05). The relative indirect effect explained 57.33% (0.43/0.75) of the relative total effect (Figure 1). The results supported H5.

Supplementary Mediation Analysis. Given that the recalling and writing task in this study incorporated nature-related elements, we sought to control for potential confounding effects of these ‘natural’ factors. To isolate the distinct influence of awe, as well as to further examine the mediating role of state connection to nature, we conducted a supplementary mediation analysis. In this analysis, we used the awe score as the independent variable, while maintaining the same mediator, dependent variable, and control variables as in the previous mediation analysis. The results showed that the indirect effect of the state connection to nature was significant, with a 95% Bootstrap confidence interval of [0.06, 0.18] (excluding 0); and this effect had an effect size of 0.11 (*SE* = 0.03). After controlling for the indirect effect, the direct effect remained significant (effect size: 0.09, *p* < 0.05); the total effect of awe score was also significant (effect size: 0.20, *p* < 0.001). These results indicated that the level of awe emotion induced by memories was the main factor influencing intention to consume ecotourism. Meanwhile, the results verified the mediating mechanism of state connections to nature in this relationship.

## 4. Experiment 2: Awe and the Intention of PB in the Future

### 4.1. Method

#### 4.1.1. Participants

Participants were recruited from two colleges in Xi’an, a city located in the northwest region of China. After excluding those who failed in the attention test, the final participants were 252 college students (128 males) aged 20.00 ± 1.44 years. All participants completed the experiment in the laboratory in exchange for a payment.

#### 4.1.2. Materials

The materials of Experiment 2 were almost the same as in Experiment 1 excepting for the awe-inducing manipulation and the measurement of the PB.

Dispositional Connection to Nature. As in Experiment 1, participants’ dispositional connections to nature were assessed using the INS (Schultz, 2002).

Awe-Inducing Manipulation. In Experiment 2, a video task was used to induce emotions. Participants in all three conditions wore headphones and watched different videos for approximately two minutes. As in Experiment 1, we used nature-related content for the videos in the awe induction task. While the positive awe video used clips from Planet Earth, a BBC documentary accompanied by exciting music; the threatening awe video used clips from a tornado video on Discovery Channel accompanied by tense music (Gordon et al., 2017; Liu et al., 2023). According to existing research, a floor-cleaning demonstration was shown in the video of the control condition (Rivera et al., 2020).

Manipulation Check. We used the same measurements of emotions after the awe-inducing manipulation as we did in Experiment 1. The psychometric properties of the measurements were all acceptable (Table 1 and Table S2).

The State of Connectedness to Nature. We used the state version of the CNS as we did in Experiment 1 (Mayer et al., 2009). The psychometric properties of the scale were all acceptable (Table 1 and Table S2).

The Intention of Pro-Environmental Behavior. According to existing research, we measured participants’ action intentions in the future on several typical private-sphere pro-environmental behaviors (PB). The scale includes five items, such as “I intend to buy environmentally friendly products in future” (Polonsky et al., 2014; see Table S2). Each item was assessed using a 7-point Likert scale, ranging from “1—strongly disagree” to “7—strongly agree”. The average score was taken as the level of the participants’ intention of future PB. The psychometric properties of the scale were acceptable (Table 1 and Table S2).

Demographic variables. Finally, demographic information was collected, including participants’ sex, age, and college.

#### 4.1.3. Procedure

Participants provided informed consent after arriving at the lab. They were first informed to complete the INS scale, and were then randomly assigned into different conditions to watch emotion-inducing videos (positive awe, threatening awe, and neutral emotion). They were then informed to complete the manipulation check of the emotion-inducing task and the state CNS. Thereafter, all participants were asked to report the intention to conduct several typical private-sphere pro-environmental behaviors in the future. Lastly, participants completed a demographic questionnaire.^2^

### 4.2. Results

Manipulation Check. Consistent with the approach in Experiment 1, a one-way ANOVA was conducted to compare emotions induced by different videos across conditions. As seen in Table 3, the results indicated that the video-watching task effectively elicited different valences of awe.

Preliminary Analysis. ANCOVAs were conducted to compare the state CNS and PB after inducing different emotions across conditions. We used participants’ sex, university and initial INS as covariates in the present analysis and further analysis. As shown in Table 3, both awe groups scored higher levels of state CNS than the control group. The results support H3 and H4. Regarding the intention to engage in future PB, the score of the positive awe group was significantly higher than that of the control group. Although the score of the threatening awe group was slightly higher than that of the control group, there was no significant difference between the two groups. The results support H1 but not H2.

Mediation Analysis. Rucker et al. (2011) and Hayes (2009) suggest that significant indirect effects can emerge even in the absence of a significant total effect. In light of this, although our preliminary analysis did not reveal a significant difference between the threatening awe and control groups in the intention of PB, we still conducted a mediation analysis. The mediation analysis followed the same procedure as in Experiment 1, except that sex, school, and dispositional nature connectedness (INS) were control variables, and the intention of PB was the dependent variable.

The results of the omnibus test showed that the total effect was significant (*F*(2, 246) = 4.97, *p* < 0.01); however, the direct effect was not (*F*(2, 245) = 0.99, *p* > 0.05). The omnibus mediation effect was significant (95% Bootstrap confidence interval [0.02, 0.08], excluding 0). Thus, we conducted the relative mediation analysis.

**Figure 2:**
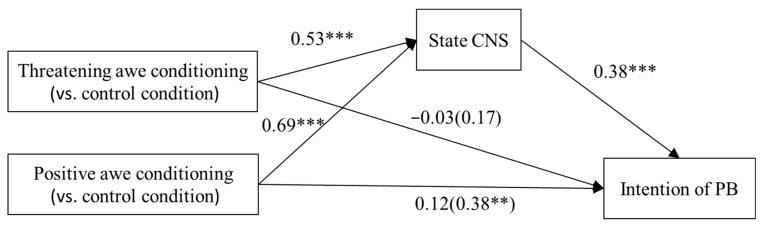
Mediation model of Experiment 2 (nonstandard results). Note. Controlling for sex, school, and INS. ** *p* < 0.01, *** *p* < 0.001.

Relative to the control condition, the relative indirect effect of the threatening awe conditioning was significant, with a 95% Bootstrap confidence interval of [0.10, 0.35], excluding 0 (see Figure 2). This effect had an effect size of 0.20 (*SE* = 0.06). However, the total effect was not statistically significant (*p* > 0.05). Additionally, after controlling for the indirect effect, the relative direct effect was also non-significant (*p* > 0.05). The results support H6. When the (relative) total effect is not statistically significant, but the (relative) indirect effect is significant; this is called the suppressing effect. This indicates that threatening awe may influence PB through other opposing mediation processes (MacKinnon et al., 2000).

Relative to the control condition, the relative indirect effect of positive awe conditioning was significant, with a 95% Bootstrap confidence interval of [0.14, 0.44], excluding 0 (see Figure 2). This effect had an effect size of 0.26 (*SE* = 0.08). Additionally, the relative total effect was significant, with an effect size of 0.38 (*p* < 0.01). After controlling for the relative indirect effect, the relative direct effect was found to be non-significant (*p* > 0.05). The relative indirect effect explained 68.42% (0.26/0.38) of the relative total effect (Figure 2). The results support H5.

Supplementary Mediation Analysis. Consistent with the approach in Experiment 1, a supplementary mediation analysis was also conducted in Experiment 2, with awe scores serving as the independent variable. The results indicated that the indirect effect of the state connection to nature was significant, with a 95% Bootstrap confidence interval of [0.03, 0.12], excluding 0; and this effect had an effect size of 0.07, (*SE* = 0.02). After controlling for the indirect effect, the direct effect remained significant (effect size: 0.15, *p* < 0.001); the total effect of awe score was also significant (effect size: 0.22, *p* < 0.001). These results implied that the level of awe emotion induced by videos was the main factor influencing the intention to engage in PB in the future. The results verified the mediating role of connection to nature in this relationship.

## 5. General Discussion

### 5.1. Effects of Awe on Connection to Nature

This paper focused on the effects of awe on connection to nature and PB through two experiments. The results of two experiments showed that both valences of awe can significantly improve the individuals’ levels of connection to nature. These results verified the relational component of awe as a self-transcendent emotion (Yaden et al., 2019).

Connection to nature is a cognitive and emotional bond between individuals and the natural environment (Tam, 2013). It is not only an effective predictor of PB, but also very important for individuals’ mental health. According to the biophilia hypothesis, connecting with nature is the basic need of human beings, which is beneficial to the physical and mental health of individuals (Kellert & Wilson, 1993). The meta-analysis showed that people who are more connected to nature also tend to have higher levels of self-reported hedonic and eudaimonic well-being (Pritchard et al., 2020). That is to say, the results of two experiments in this study showed that experiencing awe may improve one’s feeling of connection to nature, thus further having a positive effect on their happiness. This inference is consistent with the results of existing research (Liu et al., 2023; Monroy & Keltner, 2022).

In addition, according to the Attention Restoration Theory (ART; Kaplan, 1995), attention is a limited resource. Contacting with nature can alleviate the mental fatigue, restore attention resources, improve attentional level and reduce impulsive behaviors. The results of this study suggested that inducing awe might also have positive benefits on one’s executive functions, such as attention. This possibility should be explored in future research.

### 5.2. Effects of Awe on PB and the Mediating Role of Connection to Nature

The results of the main mediating analysis (using awe condition as independent variable) and the results of supplementary mediating analysis (using awe score as independent variable) were consistent across two experiments, which verified the mediating mechanism of state connection to nature in the relationship between awe (both positive and threatening) and PB. The results consistent with the self-transcendence character of awe that both positive and threatening awe could improve a sense of connection to one’s surroundings (i.e., nature) (Yaden et al., 2019). The results are also consistent with previous studies in which participants with a stronger connection to nature reported higher levels of self-engagement in PB (Whitburn et al., 2020). Furthermore, the constructionist self-theory emphasizes the importance of connection to nature, which states that if people include nature in their own identity and closely connect themselves with nature, they might engage in PB (Bragg, 1996; Tam, 2013). The results support these theoretical analyses, indicating that the self-transcendence of awe promotes a sense of connection to nature (Yaden et al., 2017) and further enhances the possibility of individuals’ PB (Tam, 2013).

Regarding the effect of awe on PB, the results of the supplementary mediating analysis showed that the total effect of awe score was significant. This indicated that the level of induced awe emotion (either positive or threatening awe) was the main factor influencing the intention to consume ecotourism and to conduct PB in the future. The results support the perspective of functionalism of awe, that experiencing awe may indicate that individuals are in a safe environment, so they are more likely to transcend themselves and further engage in pro-environmental behaviors (Chirico & Yaden, 2018).

The results of awe on the PB of the positive awe condition (vs. control condition) using ANOVAs were consistent across two experiments, while the results of threatening awe condition were not.

Using different awe-inducing tasks and different measurements of PB, the results of both experiments showed that individuals who induced positive awe, rather than neutral emotions, were more willing to engage in PB, and the connection to nature played a mediating role in this process. These findings are consistent with those of prior research that inducing positive awe can increase participants’ PB (Sun et al., 2021; Yang et al., 2018), and the feeling of connection to nature plays a mediating role in the process (Yang et al., 2018).

Although the mediating role of connection to nature has been confirmed in the threatening awe group (vs. control group) in both experiments, the results of PB in two experiments were not the same. The results of Experiment 1 showed a higher level of willingness to pay for ecotourism in the threatening awe group than the control group; while the results of Experiment 2 showed no significant differences between the threatening awe group and the control group in the intention of PB. We think there may be several possible explanations of these results.

Firstly, we speculated that these results might be due to the different levels of CNS, that is, different manipulation methods induced different levels of state CNS. To explore this possible explanation, we tried to conduct an ANCOVA to compare the state CNS of the two threatening awe groups in two experiments. In this analysis, we used participants’ INS, sex, age as covariates. The results showed that threatening awe group in Experiment 1 (*M* = 5.41, *SD* = 0.81; *F* = 7.10, *p* < 0.01, η_p_^2^ = 0.05) scored greater state CNS than the threatening awe group in Experiment 2 (*M* = 5.13, *SD* = 0.95). As the state CNS scored a bit lower, the threatening awe group in Experiment 2 might not show a significantly higher level of intention to engage in future PB than the control group. This might be the possible explanation why there was no significant difference between the threatening awe group and the control group regarding future PB in Experiment 2.

Secondly, we speculated that this may be due to the different indices of PB in the two experiments. If we regard PB as a kind of pro-social behavior, previous research on awe and pro-social behavior could also provide references for the present research.

Researchers believe there are different types of pro-social behaviors: donating money or donating time. Specifically, existing research has found that after inducing threatening awe, participants were more willing to donate money (or other types of financial support) rather than donate time to help others (Guan et al., 2019; Piff et al., 2015; Sun et al., 2021), while inducing positive awe might promote prosocial behaviors in both time and financial ways (Guan et al., 2019). These results were consistent with the results of positive awe in both experiments of this study, and with the results of threatening awe in Experiment 1. Regarding the reasons of this difference between positive and threatening awe, researchers believe that this may be because of the fear and anxiety accompanied by threatening awe could trigger an individual’s sense of time pressure and further lead to their reluctance to donate time to others (Guan et al., 2019). On the contrary, positive awe has been found to expand one’s time perception, making them feel that they have more time, so they may be more willing to spend time helping others (Rudd et al., 2012).

In Experiment 2, participants’ intention to engage in a series of PB was taken as the indicator of PB. These behaviors included various types of private-sphere behaviors which may combine financial and time support (e.g., reducing household waste; Table S2). These items may confuse the influence of threatening awe on different types of PB, resulting in no significant difference in future PB between the threatening awe and control group. It is necessary for future research to create a distinction between different kinds of PB (devoting money or time) and pay attention to other possible mechanisms, such as time pressure.

Thirdly, the results of Experiment 1 show that threatening awe promotes PB can be understood from another perspective, and that the experience of witnessing a natural disaster or an extreme weather event (to induce threatening awe) can promote PB. Previous research has found that participants who have witnessed a natural disaster or extreme weather events in their experiences are more likely to spend more money on PB themselves or support the government to do so (Falco & Corbi, 2023; López-Feldman & González, 2022). As for the reasons, extreme weather events can make the risks associated with climate change more immediate and perceptible, potentially leading to the conduct of PB (López-Feldman & González, 2022). Other researchers maintain that people may become more aware of how humans and nature interact with each other as a result of natural disasters, strengthening their bond with the natural world (connection to nature), increasing their sense of personal responsibility for the environment, and then increasing their participation in PB (Zhang et al., 2014). This explanation can be supported by our exploratory data analysis of ANCOVA, which compared the state CNS of the two threatening awe groups in two experiments. The results showed that participants who recalled their experiences of witnessing a natural disaster or extreme weather event in Experiment 1 reported higher levels of state CNS than participants who only watched an extreme weather video in Experiment 2. The results of the above research (Falco & Corbi, 2023; López-Feldman & González, 2022) were consistent with the results of threatening awe in Experiment 1, and may account for the conflicting results of those in Experiment 2.

## 6. Implications

### 6.1. Theoretical Implications

The results of this study suggest theoretical implications for both awe and PB.

First, the results of the present research suggested that both positive and threatening awe could be a potent predictor of PB in the form of financial support. According to the classification of pro-social behaviors, PB can be divided into two different forms: financial support and time support (Guan et al., 2019). While existing research of awe showed that inducing positive awe could encourage PB (Sun et al., 2021; Yang et al., 2018), this study found that inducing threatening awe could also promote the financial support of PB (i.e., willingness to pay more for ecotourism). Therefore, these findings contribute to the literature on awe and PB.

Second, while previous studies have focused more on the characteristic of weakened self-awareness of awe (e.g., Piff et al., 2015), the present research emphasized the important role of another characteristic accompanied by awe—the sense of connection (Yaden et al., 2017). The present research found that both positive and threatening awe can enhance one’s feeling of connection to nature, which is an important mediator of awe (positive and threatening) affecting PB. Moreover, an increased sense of connection to nature may also enhance one’s well-being and reduce stress (Liu et al., 2023), thus extending the positive effect of awe.

Third, this study also enriches the theories related to PB. Previous studies showed that contextual factors, attitudes toward PB, values of altruistic or egoistic, and beliefs about the behavior’s consequences can all be the predictors of PB (Ertz et al., 2016; Stern, 2000). The findings of the present research suggest that irrational factors such as emotions (i.e., awe) and affective cognition (i.e., connection to nature) may also be protective factors in environmental protection (Whitburn et al., 2020). These findings enrich the theories of PB and provide new methods in promoting PB.

### 6.2. Managerial Implications

The present study offers important managerial implications for both policy makers and ecotourism operators. Economic development should not be at the expense of the environment, and economic development and environmental protection should be placed in the same important position. At present, many countries, including the government of China, insist on implementing the strategy of sustainable development.

Ecotourism is beneficial to both the economy and environmental protection; therefore, it can be an important development direction in the tourism industry. The findings of this research indicate that feelings of awe (both positive or threatening) and the psychological connection with nature may encourage individuals to pay more money for ecotourism. Ecotourism operators and travel agents could develop communication materials that improve tourists’ feelings of awe and their psychological connection and intimacy with nature.

The findings of this study may also inform the government that improving citizens’ feelings of connection to nature could benefit the protection of nature. The results of the present research are consistent with that of previous studies (e.g., Whitburn et al., 2020)—individuals who feel more connected to nature are more likely to engage in PB. Thus, the government could invest more in public greening to make it convenient for citizens to be in contact with nature and encourage them to participate more in outdoor activities, thereby promoting their feelings of connection to nature and further PB. By making people feel closely related to nature, it would be possible to integrate environmental protection into the daily lives of all citizens and make further contributions to sustainable development.

## 7. Limitations and Future Directions

The present study has some limitations. First, given the inconsistent results of the threatening awe on PB in two experiments, future research can further clearly distinguish the two different PBs of donating time or money. Future research should investigate other potential mechanisms of awe that affect PB, such as time perception or time pressure (Rudd et al., 2012). Second, future research should use other materials (not related to nature, such as slow videos of colored water droplets colliding with milk) to induce awe to verify the results of this study (Piff et al., 2015). Third, this study focused on the emotional factors that influence PB, but of course, PB is related to many other factors, such as environmental awareness or attitude (e.g., Ramkissoon, 2020; Stern, 2000). Future research can integrate emotional factors and other theories to examine the impact of various factors more thoroughly and comprehensively. Fourth, the present research was conducted with two Chinese samples from a collectivistic culture. Individualistic and collectivistic cultures may have an impact on values, which may further influence PB (Schwartz et al., 2012; Stern, 2000). Future studies should consider validating the present results in individualistic cultures.

## 8. Conclusions

The results of this study demonstrated that positive awe can promote individuals to engage in more PB, while threatening awe can also promote it in the form of financial support (paying a premium for ecotourism). The feeling of connection to nature is one of the psychological mechanisms in these processes.

The feeling of awe is a universal human emotion that has deep roots in the evolution of human beings (Chirico & Yaden, 2018). The awe of nature should not be forgotten. Only when all mankind regains this self-transcendence emotion can we better connect with nature and protect nature.

## Figures and Tables

**Table 1:** Indices of the measurement model in Experiment 1 and 2.

Variables	α	CR	AVE
Awe (Experiment 1; Experiment 2)	0.81; 0.82	0.81; 0.83	0.59; 0.62
Fear (Experiment 1; Experiment 2)	0.85; 0.91	0.85; 0.89	0.65; 0.72
Positive affect (Experiment 1; Experiment 2)	0.84; 0.82	0.85; 0.84	0.66; 0.65
Negative affect (Experiment 1; Experiment 2)	0.52; 0.83	0.54; 0.83	0.33; 0.63
State CNS (Experiment 1; Experiment 2)	0.86; 0.87	0.90; 0.91	0.41; 0.46
Willingness to pay for ecotourism (Experiment 1)	0.86	0.86	0.56
Intention of PB (Experiment 2)	0.84	0.85	0.54

Note: The indices of the measurements of the manipulation check and state CNS include two, respectively, for the results of Experiment 1 and Experiment 2, separated by a semicolon. The index of the measurement of willingness to pay for ecotourism is for Experiment 1. The index of the measurement of intention of PB is for Experiment 2.

**Table 2:** Emotions, state CNS, and PB across groups in Experiment 1 (*n* = 166).

	Positive Awe (*n* = 58)	Threatening Awe (*n* = 55)	Control (*n* = 53)	Overall *F*	η_p_^2^
Manipulation Check					
Emotions					
Awe	5.53 (0.94) ^c^	5.21 (0.97) ^c^	3.03 (1.24) ^ab^	90.01 ***	0.53
Fear	2.60 (1.15) ^b^	5.22 (1.19) ^ac^	2.82 (1.36) ^b^	76.60 ***	0.49
Positive affect	5.10 (1.22) ^bc^	2.62 (1.24) ^ac^	3.77 (1.20) ^ab^	58.61 ***	0.42
Negative affect	1.50 (0.67) ^b^	2.47 (0.92) ^ac^	1.72 (0.52) ^b^	27.50 ***	0.25
Preliminary Analysis					
State CNS	5.73 (0.60) ^bc^	5.41 (0.81) ^ac^	4.74 (0.85) ^ab^	24.05 ***	0.23
Willingness to pay for ecotourism	5.63 (0.84) ^c^	5.53 (0.85) ^c^	4.77 (0.99) ^ab^	11.61 ***	0.13

Note. Standard deviations are shown in parentheses. The preliminary analysis controlled for sex, age, monthly income, and INS. ^a^ Mean is different from the positive awe condition. ^b^ Mean is different from the threatening awe condition. ^c^ Mean is different from the control condition at *p* < 0.05. *** *p* < 0.001.

**Table 3:** Emotions, state CNS, and PB across groups in Experiment 2 (*n* = 252).

	Positive Awe (*n* = 86)	Threatening Awe (*n* = 83)	Control (*n* = 83)	Overall *F*	η_p_^2^
Manipulation Check					
Emotions					
Awe	4.36 (1.16) ^c^	4.31 (1.16) ^c^	2.42 (1.20) ^ab^	73.91 ***	0.37
Fear	1.62 (0.99) ^b^	3.34 (1.46) ^ac^	1.45 (0.82) ^b^	72.51 ***	0.37
Positive affect	5.03 (1.15) ^bc^	3.10 (1.20) ^a^	3.57 (1.47) ^a^	52.77 ***	0.30
Negative affect	1.34 (0.61) ^b^	2.05 (1.19) ^ac^	1.17 (0.43) ^b^	28.06 ***	0.18
Preliminary Analysis					
State CNS	5.34 (0.70) ^c^	5.13 (0.95) ^c^	4.67 (0.88) ^ab^	17.02 ***	0.12
Intention of PB	6.07 (0.74) ^c^	5.83 (0.91)	5.68 (0.84) ^a^	4.48 *	0.04

Note. Standard deviations are shown in parentheses. The preliminary analysis controlled for sex, school, and INS. ^a^ Mean is different from the positive awe condition. ^b^ Mean is different from the threatening awe condition. ^c^ Mean is different from the control condition at *p* < 0.05. * *p* < 0.05, *** *p* < 0.001.

## Data Availability

The datasets analyzed during the current study are not publicly available due to the principle of protection of privacy but are available from the corresponding author upon reasonable request.

## Notes

1 Participants also reported a sense of connection with humanity and their attitudes toward hierarchy and inequality among groups (Social Dominance Orientation Scale; Ho et al., 2015). This part of the data was not related to this study; therefore, it was not discussed further.

2 Participants also reported how small and powerless they felt and completed the Satisfaction with Life Scale (Diener et al., 1985) and the Flourishing Scale (Diener et al., 2010) according to their feelings at that moment. This part of the data has been discussed in another paper (Liu et al., 2023).

## Supplementary Materials

The following supporting information can be downloaded at: https://www.mdpi.com/article/10.3390/bs15050686/s1, The Instructions of the Awe-Inducing Manipulation in Experiment 1; Table S1: Socio-demographic Profiles of Participants in Experiment 1 (n = 166); Table S2: Items and Factor Loadings of the Measurement Instruments in Experiment 1 and 2.
