# Supplementary material for: Influence of Positive and Threatening Awe on Pro-Environmental Behavior: The Mediating Role of Connection to Nature
Source: Behav Sci (Basel). 2025 May 16;15(5):686. doi: 10.3390/bs15050686 (PMC12109501; doi:10.3390/bs15050686)
Supplement: Supplementary file 1 [file behavsci-15-00686-s001.zip › behavsci-3494371-supplementary.pdf]

Supplementary Materials

Influence of Positive and Threatening Awe on Pro-Environmental Behavior: The Mediating Role of Connection to Nature

1. The Instructions of the Awe-Inducing Manipulation in Experiment 1

Positive awe conditioning: "Awe is a strong feeling that causes people to experience both respect and fear. When we are faced with vast and beautiful natural scenery or wonders, such as beautiful mountains, vast vistas of huge waterfalls, we often feel awe..."

Threatening awe conditioning: "Awe is a strong feeling that causes people to experience both respect and fear. When we are faced with huge and horrible natural disasters, such as earthquakes, volcanic eruptions, mudslides, tsunamis, typhoons, floods, we often feel awe..."

Control conditioning: "Please recall your last shopping experience in the supermarket and try to recall how you got to the supermarket from home, what the specific route was, and what other people in the supermarket were doing."

Participants assigned to the two awe conditions were also presented with an image of the awe example (Northern Lights or thunderstorm cell) on the webpage to facilitate the recall of their memories[1].

Table S1. Socio-demographic Profiles of Participants in Experiment 1 (n=166).

Variables	Categories	Frequency	Percent (%)
Sex	Male	82	49.4
	Female	84	50.6
Age	≤20	11	6.6
	21–30	73	44.0
	31–40	67	40.4
	41–50	13	7.8
	≥51	2	1.2
Education	High school or below	7	4.2
	Bachelor's degree	141	84.9
	Master's degree or above	18	10.8
Monthly Income (CNY)	≤4000	28	16.9
	4000–7000	60	36.1
	7000–10000	43	25.9
	＞10000	35	21.1
Location*	Guangdong Province	24	14.5
	Jiangsu Province	17	10.2
	Beijing	16	9.6
	Shanghai	16	9.6
	Zhejiang Province	11	6.6
	Other Locations**	82	49.4
Employment	Student	19	11.4
	Working	134	80.7
	Others	13	7.8

Note: *Location represents where more than 10 participants currently live. **Other locations includes 21 other provinces, autonomous regions, or municipalities in China other than the five listed above.

[1] The images of the awe example (Northern Lights and thunderstorm cell) are available from the corresponding author upon reasonable request.

Table S2. Items and Factor Loadings of the Measurement Instruments in Experiment 1 and 2.

Variables	Items	Factor loadings
**Awe (Experiment 1; Experiment 2)**	awe	0.600***; 0.618***
	wonder	0.900***; 0.855***
	amazement	0.773***; 0.867***
**Fear (Experiment 1; Experiment 2)**	fear	0.872***; 0.940***
	anxiety	0.749***; 0.771***
	nervousness	0.796***; 0.831***
**Positive Emotion (Experiment 1; Experiment 2)**	amusement	0.718***; 0.527***
	joy	0.952***; 0.939***
	warmth	0.746***; 0.891***
**Negative Emotion (Experiment 1; Experiment 2)**	anger	0.492***; 0.795***
	sadness	0.850***; 0.975***
	shame	0.188*; 0.566***
**State CNS (Experiment 1; Experiment 2)**	1. Right now I'm feeling a sense of oneness with the natural world around me.	0.638***; 0.557***
	2. At the moment, I'm feeling that the natural world is a community to which I belong.	0.619***; 0.630***
	3. I presently recognize and appreciate the intelligence of other living organisms.	0.668***; 0.696***
	4. At the moment, I don't feel connected to nature.	0.727***; 0.799***
	5. At the moment, I can imagine myself as part of the larger cyclical process of living.	0.616***; 0.640***
	6. At this moment, I'm feeling a kinship with animals and plants.	0.587***; 0.649***
	7. Right now, I feel as though I belong to the earth just as much as it belongs to me.	0.730***; 0.712***
	8. Right now, I am feeling deeply aware of how my actions affect the natural world.	0.647***; 0.656***
	9. Presently, I feel like I am part of the web of life.	0.724***; 0.854***
	10. Right now, I feel that all inhabitants of earth, human and nonhuman, share a common life force.	0.642***; 0.849***
	11. At the moment, I am feeling embedded within the broader natural world, like a tree in a forest.	0.765***; 0.839***
	12. When I think of humans' place on earth right now, I consider them to be the most valuable species in nature.	0.306**; 0.191*
	13. Presently, I am feeling like I am only a part of the natural world around me, and that I am no more important than the grass on the ground or the birds in the trees.	0.465***; 0.394***
**Willingness to pay for ecotourism (Experiment 1)**	1. How willing would you be to go on a more expensive holiday to reduce pollution?	0.857***
	2. How willing would you be to financially support ecotourism projects?	0.714***
	3. How willing would you be to pay more for your holiday if you knew the added cost paid for a better environment?	0.765***

	4. How willing would you be to pay more for your holiday today in exchange for possibly better tourism experiences in the future?	0.673***
	5. How willing would you be to pay more for ecotourism as opposed to “regular” tourism?	0.720***
**Intention of PB (Experiment 2)**	1. I intend to buy environmentally friendly products in the future.	0.589***
	2. I intend to buy more organic food in the future.	0.739***
	3. I intend to reduce household waste in the future.	0.800***
	4. I intend to use products made from recycled material whenever possible.	0.704***
	5. I intend to use more public transportation in the future.	0.813***

Note: The indices of the measurement of emotions and state CNS include two, respectively for the results of Experiment 1 and Experiment 2, separated by a semicolon. The index of the measurement of willingness to pay for ecotourism is for Experiment 1. The index of the measurement of intention of PB is for Experiment 2. *$p < .05$, **$p < .01$, ***$p < .001$.
